# Intravenous Opioid Administration During Mechanical Ventilation and Use After Hospital Discharge

**DOI:** 10.1001/jamanetworkopen.2024.17292

**Published:** 2024-06-14

**Authors:** Laura C. Myers, Lauren Soltesz, Nicholas Bosch, Kathleen A. Daly, Ycar Devis, Justin Rucci, Jennifer Stevens, Hannah Wunsch, S. Reza Jafarzadeh, Cynthia I. Campbell, Vincent X. Liu, Allan J. Walkey

**Affiliations:** 1 The Permanente Medical Group, Kaiser Permanente Northern California, Oakland; 2 Division of Research, Kaiser Permanente Northern California, Oakland; 3 Department of Medicine, Boston University Chobanian & Avedisian School of Medicine, Boston, Massachusetts; 4 Beth Israel Deaconess Medical Center, Boston, Massachusetts; 5 Sunnybrook Research Institute, Toronto, Ontario, Canada; 6 Department of Psychiatry and Behavioral Sciences, University of California, San Francisco; 7 Division of Health Systems Science, Department of Medicine, University of Massachusetts Chan Medical School, Worcester

## Abstract

**Importance:**

Guidelines recommend an analgesia-first strategy for sedation during mechanical ventilation, but associations between opioids provided during mechanical ventilation and posthospitalization opioid–related outcomes are unclear.

**Objective:**

To evaluate associations between an intravenous opioid dose received during mechanical ventilation and postdischarge opioid–related outcomes in medical (nonsurgical) patients.

**Design, Setting, and Participants:**

This retrospective cohort study evaluated adults receiving mechanical ventilation lasting 24 hours or more for acute respiratory failure and surviving hospitalization. Participants from 21 Kaiser Permanente Northern California hospitals from January 1, 2012, to December 31, 2019, were included. Data were analyzed from October 1, 2020, to October 31, 2023.

**Exposures:**

Terciles of median daily intravenous fentanyl equivalents during mechanical ventilation.

**Main Outcomes and Measures:**

The primary outcome was the first filled opioid prescription in 1 year after discharge. Secondary outcomes included persistent opioid use and opioid-associated complications. Secondary analyses tested for interaction between opioid doses during mechanical ventilation, prior opioid use, and posthospitalization opioid use. Estimates were based on multivariable-adjusted time-to-event analyses, with death as a competing risk, and censored for hospice or palliative care referral, rehospitalization with receipt of opioid, or loss of Kaiser Permanente plan membership.

**Results:**

The study included 6746 patients across 21 hospitals (median age, 67 years [IQR, 57-76 years]; 53.0% male). Of the participants, 3114 (46.2%) filled an opioid prescription in the year prior to admission. The median daily fentanyl equivalent during mechanical ventilation was 200 μg (IQR, 40-1000 μg), with terciles of 0 to 67 μg, more than 67 to 700 μg, and more than 700 μg. Compared with patients who did not receive opioids during mechanical ventilation (n = 1013), a higher daily opioid dose was associated with opioid prescriptions in the year after discharge (n = 2942 outcomes; tercile 1: adjusted hazard ratio [AHR], 1.00 [95% CI, 0.85-1.17], tercile 2: AHR, 1.20 [95% CI, 1.03-1.40], and tercile 3: AHR, 1.25 [95% CI, 1.07-1.47]). Higher doses of opioids during mechanical ventilation were also associated with persistent opioid use after hospitalization (n = 1410 outcomes; tercile 3 vs no opioids: odds ratio, 1.44 [95% CI, 1.14-1.83]). No interaction was observed between opioid dose during mechanical ventilation, prior opioid use, and posthospitalization opioid use.

**Conclusions and Relevance:**

In this retrospective cohort study of patients receiving mechanical ventilation, opioids administered during mechanical ventilation were associated with opioid prescriptions following hospital discharge. Additional studies to evaluate risks and benefits of strategies using lower opioid doses are warranted.

## Key Points

**Question:**

Are the administration of intravenous opioids during mechanical ventilation and the dose given associated with opioid use following hospitalization in medical (nonsurgical) patients?

**Findings:**

In this cohort study of 6746 critically ill patients who received mechanical ventilation across 21 hospitals, patients receiving higher doses of intravenous opioids during mechanical ventilation had significantly increased risks of posthospitalization opioid use.

**Meaning:**

The findings of this study suggest that intravenous opioids administered during critical illness may be associated with opioid use after hospital discharge and that additional studies of intensive care unit opioid stewardship are needed.

## Teaser

This cohort study evaluates whether intravenous opioids administered during mechanical ventilation are associated with opioid use following hospital discharge among medical (nonsurgical) patients.

## Introduction

Drug overdose deaths quintupled in the US between 1999 and 2020, with nearly 75% of overdose deaths in 2020 related to an opioid.^1^ The rapid increase in opioid overdose led to the declaration of a nationwide public health emergency in the US in 2017. Since the emergency declaration, the rate of opioid deaths in the US has continued to rise,^1^ with more than 80 000 opioid overdose deaths in 2021, 20% of which involved prescription opioids.^2^ Prescription opioids may not only be associated with overdose deaths but may also be associated with the use of nonprescription opioids with additional high risks.^3^

Several studies have examined the role of opioids administered in acute care settings within the broader context of the opioid crisis.^4,5,6,7^ For example, high-potency opioids administered in the operating room may paradoxically increase postoperative pain through rapidly induced tolerance^8^ and may lead to administration of more opioid in the postoperative period. Similarly, short-term administration of opioids in the emergency department setting may impact long-term use of opioids in the outpatient setting.^9^ However, studies have not rigorously investigated associations between opioids administered in the intensive care unit (ICU) setting during mechanical ventilation and posthospitalization opioid use.

Several factors may increase concerns that opioids prescribed during mechanical ventilation may be an important factor increasing posthospitalization opioid risks. First, critical care practice guidelines recommend high-potency intravenous opioids as the first-line sedatives for discomfort during mechanical ventilation for critically ill patients.^10^ Second, a prior study found an association between receipt of mechanical ventilation and posthospitalization opioid use^11^ but did not specifically examine the role of opioids prescribed during mechanical ventilation in the increased risk of opioid use after critical illness. Thus, we used multicenter longitudinal data from a large integrated health system, with the ability to identify prior opioid use, opioid-related diagnoses, and robust clinical information from hospitalizations of patients who received mechanical ventilation in the ICU, to evaluate the hypothesis that higher doses of intravenous opioid administered during mechanical ventilation would be associated with increased risk for opioid prescriptions after hospital discharge.

## Methods

### Study Population

This cohort study included patients hospitalized within the Kaiser Permanente Northern California (KPNC) system. It was approved by the KPNC Institutional Review Board with a waiver of informed consent because it was not practical to obtain it. The KPNC system is a large integrated health care delivery system with an electronic health record that includes highly granular and longitudinal data, capturing vital signs, laboratory values, medication administrations, and filled prescriptions across the inpatient and outpatient settings for over 4.4 million members (almost 30% of the population in Northern California). There is generally low turnover among KPNC members, with fewer than 10% lost to follow-up in 1 year. The members of KPNC are diverse and representative of Northern California’s insured population.^12^ The KPNC data source contains time-stamped administrations of all inpatient medications, including opioids. The study followed the Strengthening the Reporting of Observational Studies in Epidemiology (STROBE) reporting guideline.

**Figure 1.**
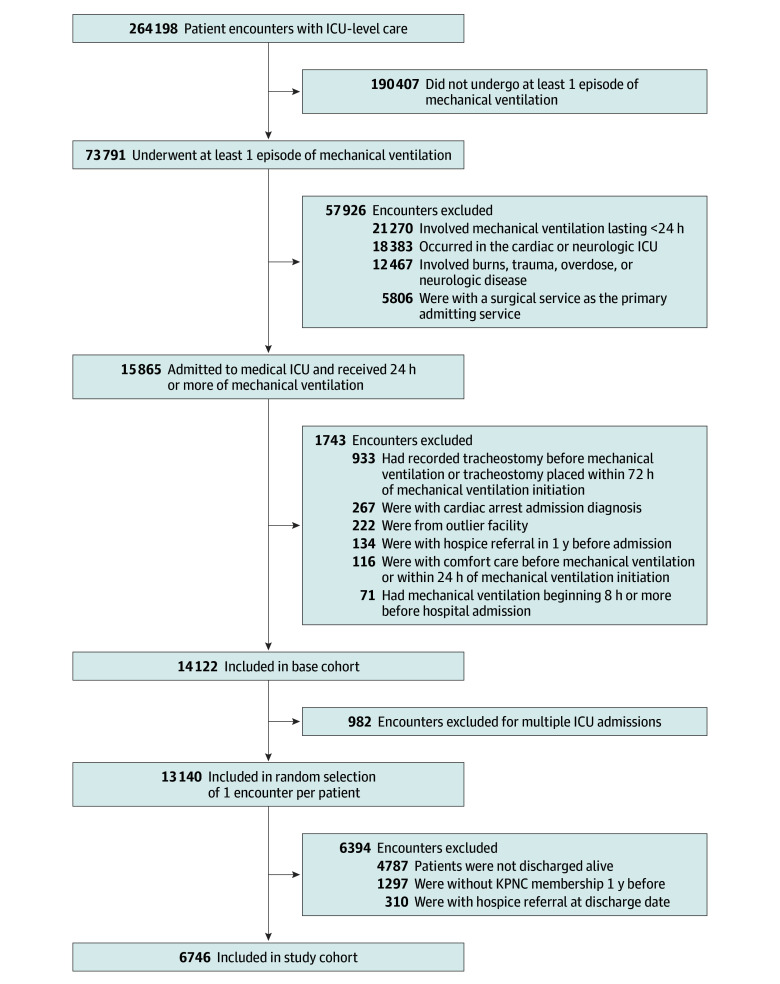
Patient Flow Diagram Patients were adults aged 18 years or older and receiving care between January 1, 2012, and December 31, 2019. ICU indicates intensive care unit; KPNC, Kaiser Permanente Northern California.

The study cohort included adults aged 18 years or older who were admitted to an ICU within 1 of KPNC’s 21 hospitals between January 1, 2012, and December 31, 2019. Patients were included if they survived to discharge and underwent an episode of mechanical ventilation that began within 8 hours of admission and lasted at least 24 hours (Figure 1). We required that patients had continuous KPNC membership for at least 1 year prior to the index hospitalization to capture important potential confounding variables (eg, filled opioid prescription) in the prehospitalization period. Race and ethnicity categories included African American or Black, Asian, Hispanic or Latinx, White, and other (Alaska Native, American Indian, Asian Pacific Islander, Native Hawaiian, multiple races or ethnicities, and unknown) and were self-identified using standardized categories from the electronic health records. Race and ethnicity were included in the study to describe the diversity of the sample of patients.

To identify a general medical (not surgical) population more likely to receive opioids for analgesia and/or sedation than for postoperative pain, we excluded patients admitted to cardiothoracic or neurosurgical specialty ICUs; those who had a primary diagnosis of cardiac arrest, burn, trauma, stroke, or intracranial hemorrhage; and those who were admitted under a primary surgical service, even if physically located within a medical ICU. . To identify patients with acute respiratory failure (vs chronic), we excluded patients who had a tracheostomy recorded either prior to mechanical ventilation or within 72 hours of initiation of mechanical ventilation. We excluded patients who had a hospice referral within 1 year prior to admission or during the hospitalization or had a comfort care order before initiation of mechanical ventilation or within 24 hours of initiation of mechanical ventilation because patients at the end of life are more likely to receive opioid medications. Among patients with multiple hospitalizations, we chose 1 encounter per patient randomly so that patient encounters in the dataset could be considered independent observations.

### Exposure

The primary exposure was median daily intravenous fentanyl equivalents (MDFEs) that could occur either through continuous opioid infusions (continuous infusion) or intermittent administrations (boluses) during the first episode of mechanical ventilation of the hospitalization. We focused on intravenous opioids, the recommended route of administration in mechanical ventilation, because they have been shown to change opioid receptors in the brain quickly and act independently of gut absorption.^10,13^ Fentanyl equivalents were used because fentanyl is the most commonly used opioid in the ICU during mechanical ventilation in the KPNC system and thus are widely understood by the intensivist community. Use of MDFEs was chosen as the exposure because patients undergo mechanical ventilation for differing lengths of time. MDFEs were calculated by starting with the first hour of mechanical ventilation, summing the total dose of fentanyl equivalents given in each 24-hour period of mechanical ventilation, and taking the median of summed fentanyl dose equivalents over all days of mechanical ventilation. Doses of hydromorphone and morphine were converted to fentanyl using the following equivalencies: 100 μg of fentanyl = 1.5 mg of hydromorphone = 10 mg of morphine.^14^ We only counted doses administered during the first 21 days of mechanical ventilation, until initiation of comfort care, or until tracheostomy placement because we were interested in doses administered during acute respiratory failure (ie, not chronic respiratory failure or for the purpose of comfort care). The recording of opioid administrations was based on medication administration records in the electronic health record. The accuracy of the hourly recording of opioid administration data was confirmed (by L.C.M.) using a 30-patient front-end audit comparing the raw data output with the electronic chart. Details of data cleaning and an example of calculating the MDFE with sample data are available in eTable 1 in Supplement 1. We categorized MDFEs into terciles to balance the ability to capture nonlinear associations with the outcome, adequately power group comparisons, and allow for clinically interpretable findings of dose ranges associated with the outcome.^15^ The reference group was patients who did not receive an intravenous opioid during their episode of mechanical ventilation.

In sensitivity analyses to evaluate robustness of results to different dose categories, we categorized the exposure of MDFEs into quartiles. Given that there is little precedent using MDFEs in the literature, we also examined the exposure in the following 2 ways other than with MDFEs: (1) the cumulative intravenous opioid in fentanyl equivalents during the entire hospitalization, including subsequent episodes of mechanical ventilation and administrations in any inpatient location including on the general medical ward and (2) the highest hourly dose of intravenous opioid administered during mechanical ventilation.

### Outcome

Given risks associated with prescription opioid use,^2^ the primary outcome was the first filled opioid prescription in the year after discharge. Outpatient pharmacy data at KPNC are nearly complete, as prescription medications are provided to members at affiliated pharmacies at a lower cost compared with non-KPNC commercial pharmacies. Each prescription is marked with the date that it was picked up by the patient.

We examined several secondary outcomes: (1) a health care encounter with an *International Classification of Diseases, Ninth Revision* or an *International Statistical Classification of Diseases and Related Health Problems, Tenth Revision* diagnosis code for opioid-related use, abuse, or overdose that occurred in either the inpatient or outpatient setting (see eTable 2 in Supplement 1 for diagnosis codes^16^ that were chosen based on previous studies^17,18^), (2) any opioid prescription filled in the 30 days after discharge, and (3) persistent opioid use over 1 year, defined as a ratio of the number of opioid prescriptions to the number of months of KPNC health care plan enrollment of 0.8 or more, based on previous studies in the perioperative space.^19,20,21^ We also examined the overlap between the definition of persistent opioid use chosen for our study and other definitions, including 10 or more prescriptions in the 1 year postdischarge period or 3 or more months of prescriptions filled.

### Risk Adjustment Variables

Confounders included sociodemographic variables (age, sex, self-identified race and ethnicity using standardized categories from the electronic health record, body mass index, and neighborhood deprivation index), smoking status, comorbidities (unweighted Charlson Comorbidity Index score sum looking back 1 year; scores range from 0 to 37, with higher scores indicating more comorbidity burden), selected individual comorbidities in the Charlson Comorbidity Index, variables associated with opioid use in the 1 year prior (previous filled opioid prescription or previous diagnosis code for opioid use, abuse, or overdose), and a previous diagnosis code for chronic pain.^22^ Confounders from the index hospitalization included principal discharge diagnosis, severity of acute illness (Sequential Organ Failure Assessment) scores in the first 24 hours of mechanical ventilation,^23^ median of the highest daily Sequential Organ Failure Assessment scores during the first 21 days of mechanical ventilation, length of hospital stay, presence of limitation of life-support interventions, number of episodes of mechanical ventilation during the index hospitalization, need for invasive monitoring (arterial or central line), and presence of a chest tube. eFigure 1 in Supplement 1 is a directed acyclic graph showing there are no collider or mediator associations. Pain was not an adjusted confounder because it is a potential mediator.

### Statistical Analysis

We used descriptive statistics for baseline characteristics of patients (median with IQR for continuous variables and number with percentage for categorical variables). Data were analyzed from October 1, 2020, to October 31, 2023.

The primary outcome was a cause-specific Cox proportional hazards regression analysis of the first filled opioid prescription in the 1 year after discharge. In addition to adjusting for confounders, we treated death as a competing risk and censored patients who received a referral to hospice or palliative care, received an inpatient opioid during a subsequent hospitalization, or were lost during follow-up due to KPNC membership discontinuation.

We performed several secondary analyses, including cause-specific Cox proportional hazards regression of an encounter for the outcome of opioid use, abuse, or overdose by diagnosis code and logistic regression for any opioid prescription filled within 1 year of discharge, any opioid prescription filled in 30 days, and persistent opioid use over 1 year. We reported effect sizes expressed as both the unadjusted and adjusted hazard ratio (HR) or odds ratio (OR), where appropriate, along with 95% CIs. We computed an E-value to estimate the strength of an association that would need to exist between a potential unmeasured confounder, primary exposure, and outcome that would shift the results to the null.^24^

To evaluate whether characteristics prior to hospitalization modified the dose response between MDFEs and posthospitalization opioid–related outcomes, we tested the single interaction between the primary exposure (MDFE) with the following variables, which were all assessed in the 1 year prior to hospitalization: (1) opioid-related diagnosis or filled opioid prescription, (2) palliative care referral, or (3) history of chronic pain diagnosis.

The threshold for significance was a 2-sided *P* < .05. R, version 4.0.2 (R Project for Statistical Computing) was used for analyses, and SAS, version 9.4 (SAS Institute Inc) was used to create figures. The STROBE checklist is available in eTable 3 in Supplement 1. The programming code is available in eTable 4 in Supplement 1.

## Results

A total of 73 791 patients were identified as adults with mechanical ventilation for medical acute respiratory failure and underwent at least 1 episode of mechanical ventilation during an ICU admission. Of these patients, there were 6746 (9.1%) who survived the hospitalization and met all inclusion and exclusion criteria to be included in the study (Figure 1). Table 1 shows the cohort characteristics by MDFE tercile. The median age of the cohort was 67 years (IQR, 57-76 years), and 3712 (47.0%) were female and 3574 (53.0%) were male. There were 845 patients (12.5%) who self-identified as African American or Black, 984 (14.6%) as Asian, 999 (14.8%) as Hispanic or Latinx, 3502 (51.9%) as White, and 416 (6.2%) as other race and ethnicity. There were 1013 patients in the reference group (15.0%) who did not receive opioids during mechanical ventilation (Table 1). Among patients who received an intravenous opioid (either using continuous infusion or bolus), the MDFE was 200 μg (IQR, 40-1000 μg). The number of patients in tercile 1 (MDFE dose, 0-67 μg) was 1932 (28.6%), 1890 (28.0%) in tercile 2 (MDFE dose, >67-700 μg), and 1911 (28.3%) in tercile 3 (MDFE dose, >700 μg). Of the participants, 3114 (46.2%) filled an opioid prescription in the year prior to admission.

**Table 1.** Baseline Characteristics of Patients

Characteristic	Patients, No. (%)				
	Overall (N = 6746)	No opioids (n = 1013)	Tercile 1 (n = 1932)^a^	Tercile 2 (n = 1890)^b^	Tercile 3 (n = 1911)^c^
Age at admission, median (IQR), y	67 (57 to 76)	70 (59 to 79)	70.0 (60 to 79)	68.0 (57 to 76)	62.0 (51 to 71)
Sex					
Female	3172 (47.0)	476 (47.0)	948 (49.1)	887 (46.9)	861 (45.1)
Male	3574 (53.0)	537 (53.0)	984 (50.9)	1003 (53.1)	1050 (54.9)
Race and ethnicity					
African American or Black	845 (12.5)	166 (16.4)	279 (14.4)	219 (11.6)	181 (9.5)
Asian	984 (14.6)	148 (14.6)	326 (16.9)	282 (14.9)	228 (11.9)
Hispanic or Latinx	999 (14.8)	141 (13.9)	297 (15.4)	248 (13.1)	313 (16.4)
White	3502 (51.9)	485 (47.9)	912 (47.2)	1021 (54.0)	1084 (56.7)
Other^d^	416 (6.2)	73 (7.2)	118 (6.1)	120 (6.3)	105 (5.5)
Admission body mass index, median (IQR)^e^	28.5 (24.0 to 34.9)	27.6 (23.3 to 34.7)	27.9 (23.4 to 33.8)	28.4 (24.1 to 346)	29.7 (24.8 to 36.3)
Neighborhood deprivation index, median (IQR)	−0.18 (−0.66 to 0.48)	−0.10 (−0.61 to 0.62)	−0.16 (−0.66 to 0.50)	−0.22 (−0.68 to 0.45)	−0.19 (−0.67 to 0.47)
1 y Prior to admission					
History of chronic pain diagnosis	1566 (23.2)	218 (21.5)	398 (20.6)	451 (23.9)	499 (26.1)
History of opioid use or abuse by diagnosis code^f^	192 (2.8)	18 (1.8)	39 (2.0)	64 (3.4)	71 (3.7)
History of opioid overdose^f^	66 (1.0)	7 (0.7)	14 (0.7)	27 (1.4)	18 (0.9)
At least 1 opioid prescription filled	3114 (46.2)	410 (40.5)	838 (43.4)	890 (47.1)	976 (51.1)
Palliative care referral	768 (11.4)	170 (16.8)	280 (14.9)	190 (10.0)	148 (7.7)
Smoking status					
Current	528 (7.8)	46 (4.5)	111 (5.7)	150 (7.9)	221 (11.6)
Former	2425 (35.9)	377 (37.2)	719 (37.2)	692 (36.6)	637 (33.3)
Never	2841 (42.1)	460 (45.4)	836 (43.3)	772 (40.8)	773 (40.5)
Unknown	952 (14.1)	130 (12.8)	266 (13.8)	276 (14.6)	280 (14.7)
Need for central line^g^	4766 (70.6)	592 (58.4)	1386 (71.7)	1275 (67.5)	1513 (79.2)
Need for arterial line^g^	1620 (24.0)	105 (10.4)	360 (18.6)	445 (23.5)	710 (37.2)
Presence of chest tube^g^	283 (4.2)	13 (1.3)	74 (3.8)	85 (4.5)	111 (5.8)
Mechanical ventilation duration, median (IQR), h^h^	62.0 (40.0 to 114.0)	46.0 (34.0 to 76.0)	72.0 (44.0 to 136.0)	49.0 (37.0 to 94.0)	71.0 (44.0 to 138.0)
Hospital length of stay, median (IQR), d	10.9 (6.8 to 18.3)	8.8 (5.8 to 15.2)	12.0 (7.3 to 20.0)	10.0 (6.2 to 17.2)	12.2 (7.7 to 19.8)
Unweighted Charlson Comorbidity Index score, median (IQR)^i^	3.0 (1.0 to 5.0)	4.0 (2.0 to 6.0)	3.0 (2.0 to 5.0)	3.0 (1.0 to 5.0)	2.0 (1.0 to 4.0)
Comorbidities					
Chronic kidney disease	2546 (37.7)	456 (45.0)	798 (41.3)	755 (39.9)	537 (28.1)
Chronic pulmonary disease	2871 (42.6)	441 (43.5)	822 (42.5)	831 (44.0)	777 (40.7)
Diabetes	2915 (43.2)	475 (46.9)	908 (47.0)	814 (43.1)	718 (37.6)
Peripheral vascular disease	3574 (53.0)	615 (60.7)	1133 (58.6)	1015 (53.7)	811 (42.4)
Chronic heart failure	2120 (31.4)	388 (38.3)	659 (34.1)	615 (32.5)	458 (24.0)
Cerebrovascular disease	1437 (21.3)	321 (31.7)	481 (24.9)	379 (20.1)	256 (13.4)
Myocardial infarction	1092 (16.2)	194 (19.2)	356 (18.4)	312 (16.5)	230 (12.0)
Mild liver disease	973 (14.4)	153 (15.1)	276 (14.3)	266 (14.1)	278 (14.6)
Localized malignant neoplasm	801 (11.9)	119 (11.8)	230 (11.9)	231 (12.2)	221 (11.6)
Metastatic malignant neoplasm	290 (4.3)	42 (4.2)	83 (4.3)	82 (4.3)	83 (4.4)
Dementia	331 (4.9)	94 (9.3)	135 (7.0)	78 (4.1)	24 (1.3)
Connective tissue disease	314 (4.7)	39 (3.9)	93 (4.8)	97 (5.1)	85 (4.5)
Peptic ulcer disease	200 (3.0)	38 (3.8)	62 (3.2)	52 (2.8)	48 (2.5)
Hemiplegia	387 (5.7)	84 (8.3)	156 (8.1)	87 (4.6)	60 (3.1)
Severe liver disease	189 (2.8)	37 (3.7)	61 (3.2)	46 (2.4)	45 (2.4)
Daily SOFA of MV1, median (IQR)^j^	7.0 (5.0 to 9.0)	6.5 (5.0 to 8.5)	7.0 (5.0 to 9.0)	7.0 (5.0 to 9.0)	7.0 (5.5 to 9.0)
Worst daily Pao_2_/FiO_2_ ratio during MV1, median (IQR)	205.0 (147.3 to 267.3)	225.0 (159.8 to 296.3)	215.0 (157.5 to 273.3)	202.8 (144.0 to 262.9)	188.9 (137.1 to 251.2)
Highest daily PEEP during MV1, median (IQR)	5.0 (5.0 to 8.0)	5.0 (5.0 to 6.0)	5.0 (5.0 to 7.0)	5.0 (5.0 to 7.0)	5.0 (5.0 to 8.0)
Tracheostomy during hospitalization	403 (6.0)	56 (5.5)	169 (8.7)	70 (3.7)	108 (5.7)
Limitation of code status after admission	1283 (19.0)	234 (23.1)	465 (24.1)	313 (16.6)	271 (14.2)
Time from intubation until comfort care status, median (IQR), h	200.9 (111.6 to 343.1)	212.2 (126.1 to 327.1)	247.2 (118.5 to 395.9)	197.6 (76.0 to 371.7)	144.1 (110.4 to 225.5)

Abbreviations: FiO_2_, fraction of inspired oxygen; MV1, first episode of mechanical ventilation; Pao_2_, arterial partial pressure of oxygen; PEEP, positive end expiratory pressure; SOFA, Sequential Organ Failure Assessment.

^a^ 0 to 67 μg.

^b^ More than 67 to 700 μg.

^c^ More than 700 μg.

^d^ Includes Alaska Native, American Indian, Asian Pacific Islander, Native Hawaiian, multiple races or ethnicities, and unknown.

^e^ Calculated as weight in kilograms divided by height in meters squared.

^f^ Diagnosis codes listed in eTable 2 in Supplement 1 were used for opioid use, abuse, and overdose.

^g^ Recorded until the first 21 days during the first episode of mechanical ventilation.

^h^ Refers to the first episode of mechanical ventilation during the hospitalization.

^i^Scores range from 0 to 37, with higher scores indicating more comorbities.

^j^ Scores range from 0 to 24, with higher scores indicating more organ dysfunction.

A total of 2942 patients (43.6%) experienced the primary outcome of a filled opioid prescription in the 1 year after discharge, of whom 353 (12.0%) were in the reference group, 741 (25.2%) were in tercile 1, 879 (29.9%) were in tercile 2, and 969 (32.9%) were in tercile 3. Among all participants, there were 475 (7.0%) who died or lost membership in the 1 year after discharge. The median time until the first opioid prescription was 26 days (IQR, 10-78 days). The median duration of mechanical ventilation was 62 hours (IQR, 40-114 hours) overall.

**Figure 2.**
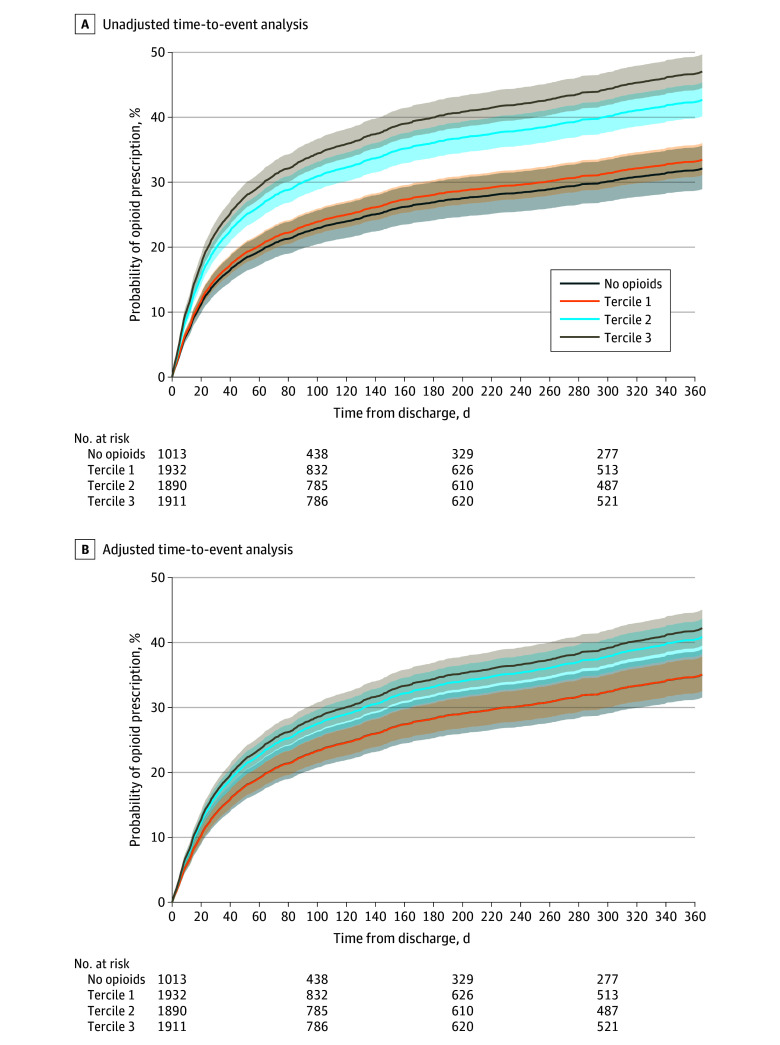
Probability of Receiving an Opioid Prescription After a Hospitalization for Mechanical Ventilation by Degree of Exposure to Opioid Tercile 1: 0 to 67 μg fentanyl equivalents; tercile 2: more than 67 to 700 μg; and tercile 3: more than 700 μg. Shaded areas represent 95% CIs.

The time to any filled opioid prescription in the 1 year after hospital discharge showed increases in the hazard of filling an opioid prescription with higher MDFE during mechanical ventilation. Compared with the reference group (no opioid receipt), the adjusted HRs were 1.00 (95% CI, 0.85-1.17) for tercile 1, 1.20 (95% CI, 1.03-1.40) for tercile 2, and 1.25 (95% CI, 1.07-1.47) for tercile 3 (Table 2). The E value was 1.61 to shift the effect estimate of tercile 3 to null and 1.47 to shift the lower 95% CI of tercile 3 to null (ie, abrogate the association). Figure 2 presents the unadjusted (Figure 2A) and adjusted (Figure 2B) cumulative event curves, showing that patients receiving higher doses of MDFEs during mechanical ventilation were more likely to fill an opioid prescription in the 1 year after discharge even after risk adjustment. eTable 5 in Supplement 1 displays the multivariable associations (β estimates) between model covariates and posthospitalization opioid use.

**Table 2.** Estimates for Opioid-Related Outcomes After Hospitalization for Mechanical Ventilation Among 6746 Patients^a^

Opioid administration^b^	AHR (95% CI)		AOR (95% CI)		
	First filled opioid prescription script in 1 y (n = 2942 outcomes)	Encounter with appropriate diagnosis (n = 258 outcomes)^c^	Persistent opioid use (n = 1410 outcomes)^d^	1 or More opioid prescriptions filled within 30 d after discharge (n = 1328 outcomes)	1 or More opioid prescriptions filled within 1 y after discharge (n = 2942 outcomes)
Tercile 1 (0-67 μg)	1.00 (0.85-1.17)	1.40 (0.75-2.59)	1.03 (0.81-1.31)	0.77 (0.61-0.97)	1.10 (0.93-1.31)
Tercile 2 (>67-700 μg)	1.20 (1.03-1.40)	1.16 (0.63-2.15)	1.50 (1.18-1.89)	1.15 (0.92-1.43)	1.36 (1.14-1.62)
Tercile 3 (>700 μg)	1.25 (1.07-1.47)	1.32 (0.72-2.42)	1.44 (1.14-1.83)	1.21 (0.97-1.51)	1.39 (1.16-1.66)

Abbreviations: AHR, adjusted hazard ratio; AOR, adjusted odds ratio.

^a^ The reference group was patients who did not receive an intravenous opioid during their episode of mechanical ventilation. Five patients had a missing value for neighborhood deprivation index or body mass index and were removed from the regression analysis. The raw outcome number is listed in each column. First opioid prescription filled within 1 year is the primary outcome column, and all other columns are secondary outcomes.

^b^ The tercile of the opioid is the median daily fentanyl equivalent of medications administered intravenously either through continuous infusion or intermittent administration during the first 21 days of mechanical ventilation until initiation of comfort care or until tracheostomy placement.

^c^ Diagnosis codes listed in eTable 2 in Supplement 1 were used for opioid use, abuse, and overdose.

^d^ The ratio of the number of prescriptions to the number of months of membership of 0.8 or more.

Higher doses of opioids during mechanical ventilation were also associated with persistent opioid use after hospitalization (n = 1410 outcomes; tercile 3 vs no opioids: OR, 1.44 [95% CI, 1.14-1.83]).

The sensitivity analyses evaluating the exposure as quartiles (instead of terciles) showed similar results (eTable 6 in Supplement 1). When we used the 2 alternative exposure definitions (cumulative fentanyl equivalent exposure over entire hospitalization and highest hourly fentanyl equivalent exposure), the findings were also similar (eTable 6 in Supplement 1).

Secondary outcomes showed similar associations as the primary outcome between increasing doses of intravenous opioids administered during mechanical ventilation and posthospitalization opioid–related outcomes (HRs and ORs range from 1.21 to 1.44 for tercile 3 vs no opioids) (Table 2). The associations with opioid-related use, abuse, or overdose and opioids prescribed within 30 days of the index hospitalization did not reach statistical significance; these outcomes had the lowest frequency of outcome events (n = 258 and n = 1328, respectively) (Table 2).

When assessing the primary outcome, none of the interaction terms were significant between the tercile of MDFE and (1) opioid-related diagnosis before hospitalization, (2) palliative care referral before hospitalization, or (3) history of chronic pain diagnosis before hospitalization (eTable 7 in Supplement 1). eFigure 2 in Supplement 1 shows the overlap of patients who met our criteria for persistent opioid use with other definitions used in the literature. Most patients meeting our definition of persistence (634 of 756 [83.9%]) also met criteria for the other 2 definitions (≥10 prescriptions in the 1 year postdischarge period or ≥3 months of prescriptions filled).

## Discussion

In this cohort study of adults receiving mechanical ventilation, we evaluated the association between intravenous opioids received during mechanical ventilation for acute respiratory failure and posthospitalization opioid-related outcomes among survivors of acute respiratory failure across 21 hospitals. Increasing doses of intravenous opioids administered during mechanical ventilation were associated with more prescription opioid use in the 1 year after discharge, including with persistent opioid use.

Our observational results are bolstered by the increase in risk with increases in opioid dose delivered during mechanical ventilation; the resilience of the findings to a moderate, theoretical, unmeasured confounding variable; the temporal proximity between the hospitalization and the filling of the first outpatient opioid prescription; and the consistency of the effect estimates across many analyses. Taken together, these findings suggest that exposure to opioid during mechanical ventilation, which may be modifiable, may influence patients’ opioid use in the long term.

Our results extend prior reports of doses of intravenous opioids used during mechanical ventilation. A 2014 Cochrane review meta-analysis of clinical trials of daily sedation interruption during mechanical ventilation showed an average dose of 300 to 1000 μg of fentanyl per day during an average of 2 to 8 days of mechanical ventilation,^25^ which was generally similar to doses observed in our study’s hospitals. However, comparisons between opioid doses across studies are difficult, especially as we required that patients survive until hospital discharge to observe posthospitalization outcomes, which likely removed more severely ill patients who potentially received higher doses of opioids.

Few prior studies have evaluated associations between opioid use during mechanical ventilation and posthospitalization outcomes. Although not examining opioid use during mechanical ventilation, a study of patients who were opioid naïve in Ontario, Canada, showed that 20% of patients who received mechanical ventilation filled an opioid prescription within 7 days of hospital discharge and 5% had persistent opioid use.^11^ Although the rates of opioid use 7 days after hospitalization within Ontario were similar to our study’s rates within 30 days, rates of persistent opioid use in our study were 4-fold higher than observed in Ontario. It is unlikely that small differences in the definition of persistent opioid use (10 or more prescriptions in 1 year vs opioids prescribed for 80% or more time alive or enrolled at KPNC) between these studies completely explains differences in rates of persistent opioid use. Although an additional study from Ontario that also evaluated opioid use after ICU admission among individuals who used opioids chronically found minimal increases in opioids prescribed after the hospitalization,^26^ our findings showed lack of effect modification by prior opioid use, suggesting that increased use of opioids after a hospitalization with mechanical ventilation may not depend on chronic opioid use prior to acute respiratory failure. Further studies evaluating differences in continuation of initial opioid prescriptions between Canada and the US are warranted to explore these national differences, which could relate to other processes of care.

A prior study showed that approximately 1% of patients had chronic opioid use in the 1 year after emergency department visits, and approximately 5% had chronic opioid use in the 90 days after minor surgical procedures.^3^ Similarly, a study of patients who were opioid naïve found that approximately 6% received new opioids in the 90 to 180 days after surgical procedures.^3^ Such findings provide context for how common persistent opioid use was in the year after mechanical ventilation for acute respiratory failure, which may be due to the combination of patients with prior opioid use and those who were opioid naïve and/or at higher risk for opioid use after critical illness. Efforts to curb chronic opioid use in non-ICU settings (such as a postoperative care setting and outpatient settings) have lowered the number of patients taking long-term opioids by up to 71%.^4,5,6,7,27,28^ Although the post-ICU setting may represent an opportunity to reduce persistent opioid use, interventions to limit opioid use in the ICU population are currently of unclear risk-benefit ratio, given the well-described negative effects of other sedatives (benzodiazepines). Additionally, while limiting opioids during mechanical ventilation may potentially reduce long-term opioid use, it is unclear what the effects of replacing opioids with other sedative or analgesic medications would have on short-term (eg, delirium or self-extubation) and long-term (eg, cognitive decline) outcomes. Moreover, for some, inadequately treated pain due to withholding appropriate opioids may have more detrimental effects both short- and long-term, which should be explored.

Although opioid use disorder following critical illness is poorly understood, survivors of acute respiratory failure requiring mechanical ventilation are noted to acquire many risk factors for opioid use disorders. For example, mood disorders and posttraumatic stress disorder are common complications following mechanical ventilation and critical illness and are consistently identified as strong risk factors of long-term prescription opioid use following initial exposure to prescription opioids.^29,30^ Understanding the mechanisms that underlie tolerance and dependence may inform the development of reversal drugs and/or raise awareness that compels clinicians to further change their prescribing behaviors.

### Strengths and Limitations

This study has several strengths. We were able to conduct our analyses accounting for prior opioid prescriptions filled, opioid-related diagnoses, and chronic pain diagnoses occurring before the index acute respiratory failure hospitalization, as well as accounting for detailed clinical information from the pre-, post-, and intrahospitalization within an integrated health system. Few data sources contain the depth and breadth needed to study the association between doses of medication administered across 21 hospitals and prescriptions filled in the 1 year after hospitalization. We estimated that a moderately strong unmeasured confounder would be required to nullify the effect estimate of the primary outcome. Although effect estimates were similar across numerous sensitivity analyses and secondary outcomes, we did not find a significant association of ICU opioid dose with posthospitalization opioid use disorders or overdose, which was potentially related to lower frequency in the outcome. The low outcome rate for diagnosis codes for opioid-related complications was in line with previous reports.^17,18^

The study also has limitations. Unmeasured confounding cannot be ruled out in observational studies. Also, the single integrated system within the US that is unusual in the integration of care for individuals, thus presenting unclear generalizability outside this health system. Illicit and unprescribed opioid use could not be captured in our analysis.

## Conclusions

The findings of this cohort study suggest that higher doses of opioids administered during mechanical ventilation may be associated with patients’ use of opioid medications following hospital discharge. These findings should motivate future studies investigating the risks and benefits of increasing opioid stewardship in the ICU setting and potentially reducing routine opioid use among patients receiving mechanical ventilation.

## Notes

Supplement 1. **eTable 1.** Details of Data Cleaning and an Example of Calculating Median Daily Fentanyl Equivalent With Sample Data **eTable 2.** Codes Used to Identify Opioid-Related Use, Abuse, Overdose **eFigure 1.** Directed Acyclic Graph **eTable 3.** STROBE Checklist **eTable 4.** Programming Code for Primary Analysis **eTable 5.** Beta Estimates of Covariates **eTable 6.** Sensitivity Analyses of Exposure as Quartiles, Cumulative Dose Over Hospitalization, and Highest Hourly Dose **eTable 7.** Additional Analyses Including Interactions Terms **eFigure 2.** Overlap of Patients Who Meet Various Definitions of Persistent Opioid Use

Supplement 2.
Data Sharing Statement
