# Supplementary material for: Intravenous Opioid Administration During Mechanical Ventilation and Use After Hospital Discharge
Source: JAMA Netw Open. 2024 Jun 14;7(6):e2417292. doi: 10.1001/jamanetworkopen.2024.17292 (PMC11179130; doi:10.1001/jamanetworkopen.2024.17292)
Supplement: Supplement 1. — eTable 1. Details of Data Cleaning and an Example of Calculating Median Daily Fentanyl Equivalent With Sample Data eTable 2. Codes Used to Identify Opioid-Related Use, Abuse, Overdose eFigure 1. Directed Acyclic Graph eTable 3. STROBE Checklist eTable 4. Programming Code for Primary Analysis eTable 5. Beta Estimates of Covariates eTable 6. Sensitivity Analyses of Exposure as Quartiles, Cumulative Dose Over Hospitalization, and Highest Hourly Dose eTable 7. Additional Analyses Including Interactions Terms eFigure 2. Overlap of Patients Who Meet Various Definitions of Persistent Opioid Use [file jamanetwopen-e2417292-s001.pdf]

Supplemental Online Content

Myers LC, Soltesz L, Bosch N, et al. Intravenous opioid administration during mechanical ventilation and use after hospital discharge. *JAMA Netw Open*. 2024;7(6):e2417292. doi:10.1001/jamanetworkopen.2024.17292

eTable 1. Details of Data Cleaning and an Example of Calculating Median Daily Fentanyl Equivalent With Sample Data
eTable 2. Codes Used to Identify Opioid-Related Use, Abuse, Overdose
eFigure 1. Directed Acyclic Graph
eTable 3. STROBE Checklist
eTable 4. Programming Code for Primary Analysis
eTable 5. Beta Estimates of Covariates
eTable 6. Sensitivity Analyses of Exposure as Quartiles, Cumulative Dose Over Hospitalization, and Highest Hourly Dose
eTable 7. Additional Analyses Including Interactions Terms
eFigure 2. Overlap of Patients Who Meet Various Definitions of Persistent Opioid Use

This supplemental material has been provided by the authors to give readers additional information about their work.

© 2024 Myers LC et al. *JAMA Network Open*.

eTable 1. Details of Data Cleaning and an Example of Calculating Median Daily Fentanyl Equivalent With Sample Data

	# Days of mechanical ventilation 1	Day 1 total (µg fentanyl)	Day 2 total (µg fentanyl)	Day 3 total (µg fentanyl)	Day 4 total (µg fentanyl)	Day 5 total (µg fentanyl)	**Median Daily Dose**
Patient 1	5	1000	2000	500	0	0	**500**
Patient 2	4	1500	2000	2000	2500	not ventilated	**2000** (only ventilated days are counted)
Patient 3	3	0	1000	500	not ventilated	not ventilated	**500**

Doses of infusion opioids are carried forward for 3 hours, meaning a fentanyl dose recorded at 10am is carried through the 11am, 12pm, and 1pm hours, unless the medication administration record (MAR) action says it was stopped/missing/due during the 3-hour period. If dose changes occurred mid hour, we carried the previous hour through until the next hour. Opioid doses were stopped at 1) first comfort care order, 2) tracheostomy timestamp or 3) after 21 days of invasive mechanical ventilation, the latter of which is the transition from acute to persistent respiratory failure in the critical care literature. Median daily fentanyl equivalent (MDFE) is calculated by summing the fentanyl equivalent for each 24h window of invasive mechanical ventilation and then taking the median across days.

© 2024 Myers LC et al. *JAMA Network Open*.

eTable 2. Codes Used to Identify Opioid-Related Use, Abuse, Overdose

For opioid use, abuse and overdose, the following codes were used:

Code	Description
ICD-9	
304	Opioid Type Dependence, Unspecified
304.01	Opioid Type Dependence, Continuous
304.02	Opioid Type Dependence, Episodic
304.03	Opioid Type Dependence, In Remission
304.7	Combinations Of Opioid Type Drug With Any Other Drug Dependence, Unspecified
304.71	Combinations Of Opioid Type Drug With Any Other Drug Dependence, Continuous
304.72	Combinations Of Opioid Type Drug With Any Other Drug Dependence, Episodic
304.73	Combinations Of Opioid Type Drug With Any Other Drug Dependence, In Remission
305.5	Opioid Abuse, Unspecified
305.51	Opioid Abuse, Continuous
305.52	Opioid Abuse, Episodic
305.53	Opioid Abuse, In Remission
965.0	Poisoning by opiates and related narcotics
965.00	Poisoning by opium, unspecified
965.01	Poisoning by heroin
965.02	Poisoning by methadone
965.09	Poisoning by other opiates and narcotics
E850	Accidental poisoning by analgesics antipyretics and antirheumatics
E850.0	Accidental poisoning by heroin
E850.1	Accidental poisoning by methadone
E850.2	Accidental poisoning by opiates and related narcotics
E935.0	Heroin causing adverse effects in therapeutic use
E935.1	Methadone causing adverse effects in therapeutic use
E935.2	Other opiates and related narcotics causing adverse effects in therapeutic use
ICD-10	
F11.10	Opioid Abuse, Uncomplicated
F11.120	Opioid Abuse With Intoxication, Uncomplicated
F11.121	Opioid Abuse With Intoxication Delirium
F11.122	Opioid Abuse With Intoxication With Perceptual Disturbance
F11.129	Opioid Abuse With Intoxication, Unspecified
F11.14	Opioid Abuse With Opioid-Induced Mood Disorder
F11.150	Opioid Abuse With Opioid-Induced Psychotic Disorder With Delusions
F11.151	Opioid Abuse With Opioid-Induced Psychotic Disorder With Hallucinations
F11.159	Opioid Abuse With Opioid-Induced Psychotic Disorder, Unspecified
F11.181	Opioid Abuse With Opioid-Induced Sexual Dysfunction
F11.182	Opioid Abuse With Opioid-Induced Sleep Disorder
F11.188	Opioid Abuse With Other Opioid-Induced Disorder
F11.19	Opioid Abuse With Unspecified Opioid-Induced Disorder

© 2024 Myers LC et al. *JAMA Network Open*.

Code	Description
F11.20	Opioid Dependence, Uncomplicated
F11.21	Opioid Dependence, In Remission
F11.220	Opioid Dependence With Intoxication, Uncomplicated
F11.221	Opioid Dependence With Intoxication Delirium
F11.222	Opioid Dependence With Intoxication With Perceptual Disturbance
F11.229	Opioid Dependence With Intoxication, Unspecified
F11.23	Opioid Dependence With Withdrawal
F11.24	Opioid Dependence With Opioid-Induced Mood Disorder
F11.250	Opioid Dependence With Opioid-Induced Psychotic Disorder With Delusions
F11.251	Opioid Dependence With Opioid-Induced Psychotic Disorder With Hallucinations
F11.259	Opioid Dependence With Opioid-Induced Psychotic Disorder, Unspecified
F11.281	Opioid Dependence With Opioid-Induced Sexual Dysfunction
F11.282	Opioid Dependence With Opioid-Induced Sleep Disorder
F11.288	Opioid Dependence With Other Opioid-Induced Disorder
F11.29	Opioid Dependence With Unspecified Opioid-Induced Disorder
F11.90	Opioid Use, Unspecified, Uncomplicated
F11.920	Opioid Use, Unspecified With Intoxication, Uncomplicated
F11.921	Opioid Use, Unspecified With Intoxication Delirium
F11.922	Opioid Use, Unspecified With Intoxication With Perceptual Disturbance
F11.929	Opioid Use, Unspecified With Intoxication, Unspecified
F11.93	Opioid Use, Unspecified With Withdrawal
F11.94	Opioid Use, Unspecified With Opioid-Induced Mood Disorder
F11.950	Opioid Use, Unspecified With Opioid-Induced Psychotic Disorder With Delusions
F11.951	Opioid Use, Unspecified With Opioid-Induced Psychotic Disorder With Hallucinations
F11.959	Opioid Use, Unspecified With Opioid-Induced Psychotic Disorder, Unspecified
F11.981	Opioid Use, Unspecified With Opioid-Induced Sexual Dysfunction
F11.982	Opioid Use, Unspecified With Opioid-Induced Sleep Disorder
F11.988	Opioid Use, Unspecified With Other Opioid-Induced Disorder
F11.99	Opioid Use, Unspecified With Unspecified Opioid-Induced Disorder
T40.0	Poisoning by, adverse effect of and underdosing of opium
T40.0X	Poisoning by, adverse effect of and underdosing of opium
T40.0X1	Poisoning by opium, accidental (unintentional)
T40.0X1A	Poisoning by opium, accidental (unintentional), initial encounter
T40.0X1D	Poisoning by opium, accidental (unintentional), subsequent encounter
T40.0X1S	Poisoning by opium, accidental (unintentional), sequela
T40.0X2	Poisoning by opium, intentional self-harm
T40.0X2A	Poisoning by opium, intentional self-harm, initial encounter
T40.0X2D	Poisoning by opium, intentional self-harm, subsequent encounter
T40.0X2S	Poisoning by opium, intentional self-harm, sequela
T40.0X3	Poisoning by opium, assault
T40.0X3A	Poisoning by opium, assault, initial encounter
T40.0X3D	Poisoning by opium, assault, subsequent encounter
T40.0X3S	Poisoning by opium, assault, sequela

© 2024 Myers LC et al. *JAMA Network Open*.

Code	Description
T40.0X4	Poisoning by opium, undetermined
T40.0X4A	Poisoning by opium, undetermined, initial encounter
T40.0X4D	Poisoning by opium, undetermined, subsequent encounter
T40.0X4S	Poisoning by opium, undetermined, sequela
T40.1	Poisoning by and adverse effect of heroin
T40.1X	Poisoning by and adverse effect of heroin
T40.1X1	Poisoning by heroin, accidental (unintentional)
T40.1X1A	Poisoning by heroin, accidental (unintentional), initial encounter
T40.1X1D	Poisoning by heroin, accidental (unintentional), subsequent encounter
T40.1X1S	Poisoning by heroin, accidental (unintentional), sequela
T40.1X2	Poisoning by heroin, intentional self-harm
T40.1X2A	Poisoning by heroin, intentional self-harm, initial encounter
T40.1X2D	Poisoning by heroin, intentional self-harm, subsequent encounter
T40.1X2S	Poisoning by heroin, intentional self-harm, sequela
T40.1X3A	Poisoning by heroin, assault, initial encounter
T40.1X3D	Poisoning by heroin, assault, subsequent encounter
T40.1X3S	Poisoning by heroin, assault, sequela
T40.1X4A	Poisoning by heroin, undetermined, initial encounter
T40.1X4D	Poisoning by heroin, undetermined, subsequent encounter
T40.1X4S	Poisoning by heroin, undetermined, sequela
T40.2	Poisoning by, adverse effect of and underdosing of other opioids
T40.2X	Poisoning by, adverse effect of and underdosing of other opioids
T40.2X1	Poisoning by other opioids, accidental (unintentional)
T40.2X1A	Poisoning by other opioids, accidental (unintentional), initial encounter
T40.2X1D	Poisoning by other opioids, accidental (unintentional), subsequent encounter
T40.2X1S	Poisoning by other opioids, accidental (unintentional), sequela
T40.2X2	Poisoning by other opioids, intentional self-harm
T40.2X2A	Poisoning by other opioids, intentional self-harm, initial encounter
T40.2X2D	Poisoning by other opioids, intentional self-harm, subsequent encounter
T40.2X2S	Poisoning by other opioids, intentional self-harm, sequela
T40.2X3	Poisoning by other opioids, assault
T40.2X3A	Poisoning by other opioids, assault, initial encounter
T40.2X3D	Poisoning by other opioids, assault, subsequent encounter
T40.2X3S	Poisoning by other opioids, assault, sequela
T40.2X4	Poisoning by other opioids, undetermined
T40.2X4A	Poisoning by other opioids, undetermined, initial encounter
T40.2X4D	Poisoning by other opioids, undetermined, subsequent encounter
T40.2X4S	Poisoning by other opioids, undetermined, sequela
T40.3	Poisoning by, adverse effect of and underdosing of methadone
T40.3X	Poisoning by, adverse effect of and underdosing of methadone
T40.3X1	Poisoning by methadone, accidental (unintentional)
T40.3X1A	Poisoning by methadone, accidental (unintentional), initial encounter
T40.3X1D	Poisoning by methadone, accidental (unintentional), subsequent encounter

© 2024 Myers LC et al. *JAMA Network Open*.

Code	Description
T40.3X1S	Poisoning by methadone, accidental (unintentional), sequela
T40.3X2	Poisoning by methadone, intentional self-harm
T40.3X2A	Poisoning by methadone, intentional self-harm, initial encounter
T40.3X2D	Poisoning by methadone, intentional self-harm, subsequent encounter
T40.3X2S	Poisoning by methadone, intentional self-harm, sequela
T40.3X3	Poisoning by methadone, assault
T40.3X3A	Poisoning by methadone, assault, initial encounter
T40.3X3D	Poisoning by methadone, assault, subsequent encounter
T40.3X3S	Poisoning by methadone, assault, sequela
T40.3X4	Poisoning by methadone, undetermined
T40.3X4A	Poisoning by methadone, undetermined, initial encounter
T40.3X4D	Poisoning by methadone, undetermined, subsequent encounter
T40.3X4S	Poisoning by methadone, undetermined, sequela
T40.4	Poisoning by, adverse effect of and underdosing of other synthetic narcotics
T40.4X	Poisoning by, adverse effect of and underdosing of other synthetic narcotics
T40.4X1	Poisoning by other synthetic narcotics, accidental (unintentional)
T40.4X1A	Poisoning by other synthetic narcotics, accidental (unintentional), initial encounter
T40.4X1D	Poisoning by other synthetic narcotics, accidental (unintentional), subsequent encounter
T40.4X1S	Poisoning by other synthetic narcotics, accidental (unintentional), sequela
T40.4X2	Poisoning by other synthetic narcotics, intentional self-harm
T40.4X2A	Poisoning by other synthetic narcotics, intentional self-harm, initial encounter
T40.4X2D	Poisoning by other synthetic narcotics, intentional self-harm, subsequent encounter
T40.4X2S	Poisoning by other synthetic narcotics, intentional self-harm, sequela
T40.4X3	Poisoning by other synthetic narcotics, assault
T40.4X3A	Poisoning by other synthetic narcotics, assault, initial encounter
T40.4X3D	Poisoning by other synthetic narcotics, assault, subsequent encounter
T40.4X3S	Poisoning by other synthetic narcotics, assault, sequela
T40.4X4	Poisoning by other synthetic narcotics, undetermined
T40.4X4A	Poisoning by other synthetic narcotics, undetermined, initial encounter
T40.4X4D	Poisoning by other synthetic narcotics, undetermined, subsequent encounter
T40.4X4S	Poisoning by other synthetic narcotics, undetermined, sequela
T40.6	Poisoning by, adverse effect of and underdosing of other and unspecified narcotics
T40.60	Poisoning by, adverse effect of and underdosing of unspecified narcotics
T40.601	Poisoning by unspecified narcotics, accidental (unintentional)
T40.601A	Poisoning by unspecified narcotics, accidental (unintentional), initial encounter
T40.601D	Poisoning by unspecified narcotics, accidental (unintentional), subsequent encounter
T40.601S	Poisoning by unspecified narcotics, accidental (unintentional), sequela
T40.602	Poisoning by unspecified narcotics, intentional self-harm
T40.602A	Poisoning by unspecified narcotics, intentional self-harm, initial encounter
T40.602D	Poisoning by unspecified narcotics, intentional self-harm, subsequent encounter
T40.602S	Poisoning by unspecified narcotics, intentional self-harm, sequela
T40.603	Poisoning by unspecified narcotics, assault
T40.603A	Poisoning by unspecified narcotics, assault, initial encounter

© 2024 Myers LC et al. *JAMA Network Open*.

Code	Description
T40.603D	Poisoning by unspecified narcotics, assault, subsequent encounter
T40.603S	Poisoning by unspecified narcotics, assault, sequela
T40.604	Poisoning by unspecified narcotics, undetermined
T40.604A	Poisoning by unspecified narcotics, undetermined, initial encounter
T40.604D	Poisoning by unspecified narcotics, undetermined, subsequent encounter
T40.604S	Poisoning by unspecified narcotics, undetermined, sequela
T40.69	Poisoning by, adverse effect of and underdosing of other narcotics
T40.691	Poisoning by other narcotics, accidental (unintentional)
T40.691A	Poisoning by other narcotics, accidental (unintentional), initial encounter
T40.691D	Poisoning by other narcotics, accidental (unintentional), subsequent encounter
T40.691S	Poisoning by other narcotics, accidental (unintentional), sequela
T40.692	Poisoning by other narcotics, intentional self-harm
T40.692A	Poisoning by other narcotics, intentional self-harm, initial encounter
T40.692D	Poisoning by other narcotics, intentional self-harm,
T40.692S	Poisoning by other narcotics, intentional self-harm, sequela
T40.693	Poisoning by other narcotics, assault
T40.693A	Poisoning by other narcotics, assault, initial encounter
T40.693D	Poisoning by other narcotics, assault, subsequent encounter
T40.693S	Poisoning by other narcotics, assault, sequela
T40.694	Poisoning by other narcotics, undetermined
T40.694A	Poisoning by other narcotics, undetermined, initial encounter
T40.694D	Poisoning by other narcotics, undetermined, subsequent encounter
T40.694S	Poisoning by other narcotics, undetermined, sequela

© 2024 Myers LC et al. *JAMA Network Open*.

eFigure 1. Directed Acyclic Graph

Minimal sufficient adjustment sets containing # episodes of MV, BMI, Comorbidities, ICU invasive procedures, LOS, Principal diagnosis, Severity of illness, age, chronic pain, code status, prior opioid use, race/ethnicity, sex, socioeconomic status, tobacco use for estimating the total effect of Opioid dose prescribed during MV on Opioid use after hospitalization:

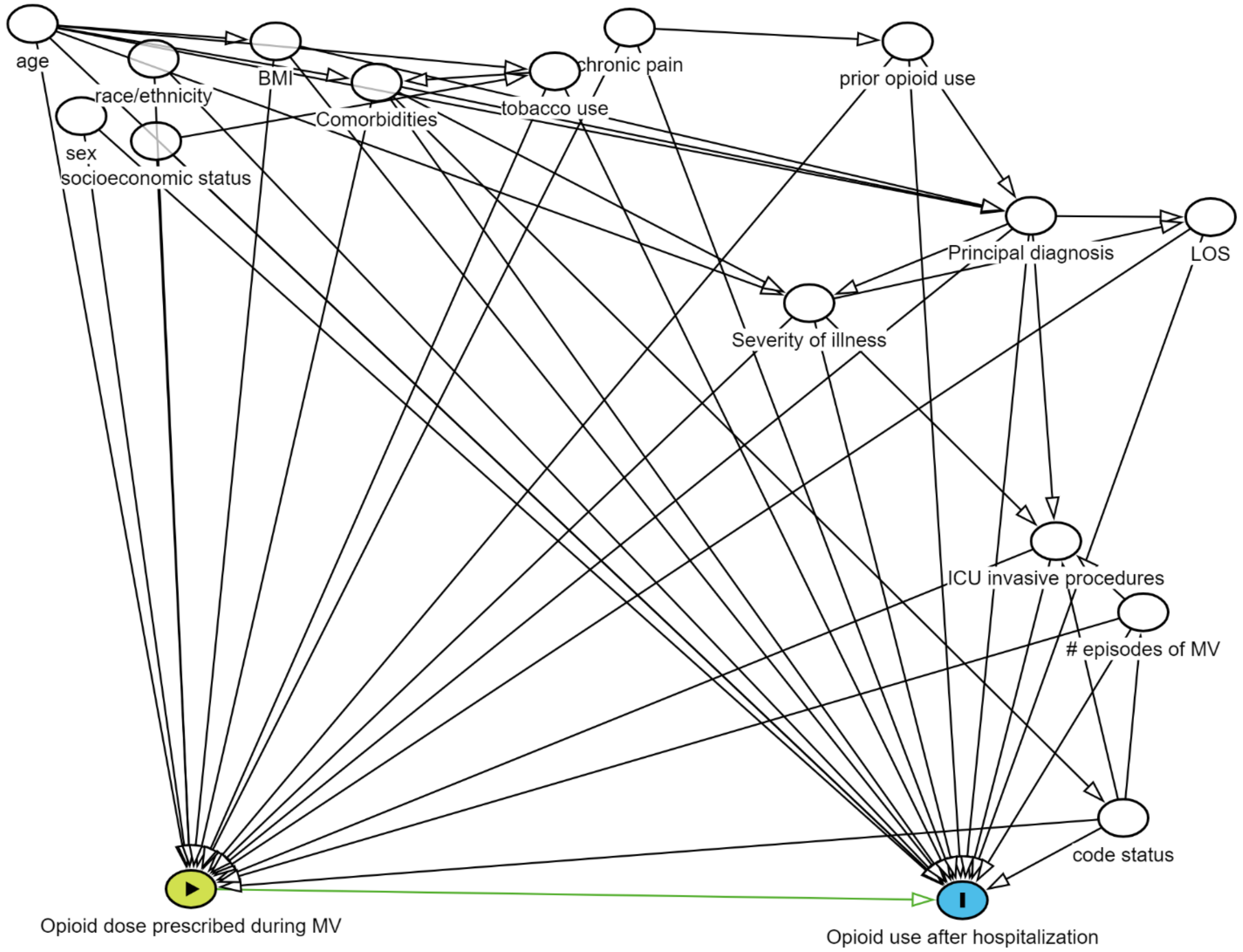

© 2024 Myers LC et al. *JAMA Network Open*.

eTable 3. STROBE Checklist

STROBE Statement—Checklist of items that should be included in reports of ***cohort studies***

	Item No	Recommendation	Page No
**Title and abstract**	1	(*a*) Indicate the study's design with a commonly used term in the title or the abstract	1
		(*b*) Provide in the abstract an informative and balanced summary of what was done and what was found	4
**Introduction**			
Background/rationale	2	Explain the scientific background and rationale for the investigation being reported	6
Objectives	3	State specific objectives, including any prespecified hypotheses	7
**Methods**			
Study design	4	Present key elements of study design early in the paper	7
Setting	5	Describe the setting, locations, and relevant dates, including periods of recruitment, exposure, follow-up, and data collection	7
Participants	6	(*a*) Give the eligibility criteria, and the sources and methods of selection of participants. Describe methods of follow-up	8
		(*b*) For matched studies, give matching criteria and number of exposed and unexposed	
Variables	7	Clearly define all outcomes, exposures, predictors, potential confounders, and effect modifiers. Give diagnostic criteria, if applicable	10
Data sources/ measurement	8*	For each variable of interest, give sources of data and details of methods of assessment (measurement). Describe comparability of assessment methods if there is more than one group	7
Bias	9	Describe any efforts to address potential sources of bias	8
Study size	10	Explain how the study size was arrived at	7,8
Quantitative variables	11	Explain how quantitative variables were handled in the analyses. If applicable, describe which groupings were chosen and why	11
Statistical methods	12	(*a*) Describe all statistical methods, including those used to control for confounding	12,14,30
		(*b*) Describe any methods used to examine subgroups and interactions	
		(*c*) Explain how missing data were addressed	
		(*d*) If applicable, explain how loss to follow-up was addressed	
		(*e*) Describe any sensitivity analyses	

© 2024 Myers LC et al. *JAMA Network Open*.

**Results**			
Participants	13*	(a) Report numbers of individuals at each stage of study—eg numbers potentially eligible, examined for eligibility, confirmed eligible, included in the study, completing follow-up, and analysed<br>(b) Give reasons for non-participation at each stage<br>(c) Consider use of a flow diagram	13
Descriptive data	14*	(a) Give characteristics of study participants (eg demographic, clinical, social) and information on exposures and potential confounders<br>(b) Indicate number of participants with missing data for each variable of interest<br>(c) Summarise follow-up time (eg, average and total amount)	13
Outcome data	15*	Report numbers of outcome events or summary measures over time	13,14
Main results	16	(*a*) Give unadjusted estimates and, if applicable, confounder-adjusted estimates and their precision (eg, 95% confidence interval). Make clear which confounders were adjusted for and why they were included<br>(*b*) Report category boundaries when continuous variables were categorized<br>(*c*) If relevant, consider translating estimates of relative risk into absolute risk for a meaningful time period	14
Other analyses	17	Report other analyses done—eg analyses of subgroups and interactions, and sensitivity analyses	15
**Discussion**			
Key results	18	Summarise key results with reference to study objectives	16
Limitations	19	Discuss limitations of the study, taking into account sources of potential bias or imprecision. Discuss both direction and magnitude of any potential bias	18
Interpretation	20	Give a cautious overall interpretation of results considering objectives, limitations, multiplicity of analyses, results from similar studies, and other relevant evidence	15,16
Generalisability	21	Discuss the generalisability (external validity) of the study results	19
**Other information**			
Funding	22	Give the source of funding and the role of the funders for the present study and, if applicable, for the original study on which the present article is based	20

*Give information separately for exposed and unexposed groups.

© 2024 Myers LC et al. *JAMA Network Open*.

eTable 4. Programming Code for Primary Analysis

```
# competing risk models in R

library(tidyverse)
library(tableone)
library(kableExtra)
library(Publish)
library(riskRegression)
library(survival)
library(DescTools)
library(gtsummary)
library(janitor)

#read in modeling dataset
modeling_data=read.csv(file.path(dataloc,
"aim3_tte_modeling_data.csv"))%>%
  mutate(IM=if_else(CENTRAL_LINE=="Y"|ARTERIAL_LINE=="Y", "Y", "N"))

modeling_data%>%select(SMOKING_STATUS, SOFA_DAY1, SOFA_MEDIAN21, IM,
CHEST_TUBE, MV_EPISODES)%>%
  tbl_summary(
    missing = "ifany",
    missing_text = "Missing")

covars=c("AGE", "GENDER", "RACE", "BMI", "NDI",
         "C_SCORE_UW", "REN", "CPD", "DIAB_ANY", "PVD", "CHD", "CVD",
"MI", "LIVD", "CANC",
         "DX_CAT", "LIMIT_CODESTATUS",
         "PALL_PRE", "OPIOID_PRE", "CHRONIC_PAIN_PRE","LOS",
         "SMOKING_STATUS",
         "SOFA_DAY1", "SOFA_MEDIAN21",
         "IM", "CHEST_TUBE", "MV_EPISODES",
         "MDFE_GRP"
)

covars_string <- paste(covars,collapse='+')

## 1. Competing risks models.
#### i. Univariate
myreg <-
paste0("CSC(Hist(time_to_event,status)~MDFE_GRP,data=modeling_data)")
fitcsc_train <- eval(parse(text = myreg))
fitcsc_train

#### ii. Multivariate
myreg <-
paste0("CSC(Hist(time_to_event,status)~",covars_string,",data=modeling_d
ata)")
fitcsc_train <- eval(parse(text = myreg))
fitcsc_train
```

© 2024 Myers LC et al. *JAMA Network Open*.

```
/* plot survival curves in SAS*/

/* import data */
PROC IMPORT
DATAFILE="~/aim3_tte_modeling_data.csv"
DBMS=csv
OUT=modeling_data replace;
run;

data Inrisks;
length MDFE_GRP $23.;
MDFE_GRP='No Opioids'; output;
MDFE_GRP='Tercile 1 (0-67 mcg)'; output;
MDFE_GRP='Tercile 2 (>67-700 mcg)'; output;
MDFE_GRP='Tercile 3 (>700 mcg)'; output;
run;

/* competing risk model univariate  */
proc phreg data=modeling_data plots(overlay=stratum)=cif;
class MDFE_GRP(ref='No Opioids') ;
model time_to_event*status(0)=MDFE_GRP/eventcode(cox)=1;
hazardratio MDFE_GRP/cl=both;
baseline covariates=Inrisks out=Pred2 CIF=_ALL_/rowid=MDFE_GRP;
output out=surv survival=s atrisk=risk/method=pl ;
run;

/*get at risk at all time periods -- for table below graph*/
proc LIFETEST data=modeling_data   plots=s(atrisk(outside maxlen=13)=0
to 400 by 1) outcif=outcif noprint;
   time time_to_event*status(0)/eventcode=1;
   strata MDFE_GRP / order=internal;
run;

/* fill in all values for time when missing */
data outcif2 (where= (conftype ne ''));
      set outcif;
      ttr=lag(time_to_event);
      atr=lag(atrisk);
      if ttr ne . then
            do i=ttr to time_to_event-1 by 1;
                  ttr=ttr+1;
                  output;
                  end;
      run;

/* join to output from phreg */
PROC SQL;
   CREATE TABLE WORK.pred3 AS
   SELECT t1.MDFE_GRP,
```

© 2024 Myers LC et al. *JAMA Network Open*.

```
          t1.time_to_event,
          t1.CIF,
          t1.StdErrCIF,
          t1.LowerCIF,
          t1.UpperCIF,
          t2.atr
      FROM WORK.PRED2 t1
           LEFT JOIN WORK.OUTCIF2 t2 ON (t1.MDFE_GRP = t2.MDFE_GRP) AND
(t1.time_to_event = t2.i);
QUIT;

ods graphics on / reset;
ODS GRAPHICS/ RESET IMAGENAME = 'unadjusted'
IMAGEFMT =PNG HEIGHT = 8in WIDTH = 11in ;
ODS LISTING GPATH = "~/Output";

/* for atrisk table, only keep values in increment of 100 */
data pred3;
      set pred3;
      if mod(time_to_event,100) ne 0 then atr=.;
      if time_to_event<=365;
      run;

ODS GRAPHICS/ RESET IMAGENAME = 'unadjusted_with_table'
IMAGEFMT =PNG HEIGHT = 8in WIDTH = 11in ;
ODS LISTING GPATH = "~/Output";
proc sgplot data=pred3 noautolegend;
band x=time_to_event lower=LowerCIF upper=UpperCIF/
fillattrs=GraphData(transparency=0.8)
group=MDFE_GRP ;
styleattrs   datacolors=(black blue green red);
series x=time_to_event y=CIF / group=MDFE_GRP  name="series"
LEGENDLABEL = 'MDFE Group'  LINEATTRS = (THICKNESS = 3 pattern=1)
attrid=attrmapping;
styleattrs datacontrastcolors=(black blue green red);
xaxis label="Days from Discharge" values=(0 to 364 by 20);
yaxis grid  label="Probability of Opioid Script"
LABELATTRS=(Color=Black Family=Arial Size=10)
VALUEATTRS=(Family=Arial Size=10) ranges=(0-.5) valuesformat=percent6.1;
xaxistable atr/x=time_to_event class=mdfe_grp title="Number At Risk"
NOMISSINGCHAR
      labelattrs=(weight=bold)
      titleattrs=(weight=bold);
keylegend "series" / title="";
title 'Unadjusted';
run;


*********************ADJUSTED****************************;
***for adjusted model need to create dummy variables for categories
first;
*adjusted;
```

© 2024 Myers LC et al. *JAMA Network Open*.

```
/*  */
/* "AGE", "GENDER", "RACE", "BMI", "NDI","C_SCORE_UW", "REN", "CPD",
"DIAB_ANY", "PVD", "CHD", "CVD", "MI", "LIVD", "CANC", */
/* "DX_CAT", "LIMIT_CODESTATUS", "PALL_PRE", "OPIOID_PRE",
"CHRONIC_PAIN_PRE","LOS",  SMOKING_STATUS, */
/*    SOFA_DAY1, SOFA_MEDIAN21, IM, CHEST_TUBE, MV_EPISODES */



data modeling_data_nomiss;
set modeling_data;
if cmiss(of _all_) then delete;
run;

/* proc contents data=modeling_data_nomiss; */
/* run; */

%macro create_indicators(input_data, target, covariates, output_data);
proc logistic
     data = &input_data
          noprint
          outdesign = indicators;
     class &covariates / param = glm;
     model &target = &covariates;
run;
data &output_data;
      merge    &input_data
               indicators (drop = Intercept &target);
run;
proc datasets
     library = work
          noprint;
     delete indicators;
run;
%mend;

%create_indicators(modeling_data_nomiss, status, GENDER RACE DX_CAT
LIMIT_CODESTATUS CHRONIC_PAIN_PRE IM CHEST_TUBE SMOKING_STATUS,
modeling_dummy);

proc means data=modeling_dummy;
var AGE GENDERM RACEBlack RACEHispanic RACEOther RACEWhite BMI NDI
C_SCORE_UW REN CPD DIAB_ANY PVD CHD CVD MI LIVD CANC
DX_CATams DX_CATarrest_bra DX_CATarrhythmia DX_CATbelly DX_CATbleed
DX_CATcancer DX_CATchf DX_CATcopd DX_CATdm_lyte DX_CATembolus
DX_CATliver DX_CATmyocardial DX_CATneuro DX_CATother DX_CATpanc
DX_CATpna DX_CATrenal DX_CATresp DX_CATseizure DX_CATsepsis
LIMIT_CODESTATUSY PALL_PRE OPIOID_PRE CHRONIC_PAIN_PREY LOS
SMOKING_STATUSCURRENT SMOKING_STATUSFORMER SMOKING_STATUSNEVER
SMOKING_STATUSUNKNOWN
SOFA_DAY1 SOFA_MEDIAN21 IMY CHEST_TUBEY MV_EPISODES;
output out=mymeans;
```

© 2024 Myers LC et al. *JAMA Network Open*.

```
run;


data Inrisks;
set mymeans;
where _STAT_="MEAN";
length MDFE_GRP $23.;
MDFE_GRP='No Opioids'; output;
MDFE_GRP='Tercile 1 (0-67 mcg)'; output;
MDFE_GRP='Tercile 2 (>67-700 mcg)'; output;
MDFE_GRP='Tercile 3 (>700 mcg)'; output;
DROP _TYPE_ _FREQ_ _STAT_;
run;

/* multivariate model */
proc phreg data=modeling_dummy plots(overlay=stratum)=cif;
class MDFE_GRP(ref='No Opioids') ;
model time_to_event*status(0)=AGE GENDERM RACEBlack RACEHispanic
RACEOther RACEWhite BMI NDI C_SCORE_UW REN CPD DIAB_ANY PVD CHD CVD MI
LIVD CANC
DX_CATams DX_CATarrest_bra DX_CATarrhythmia DX_CATbelly DX_CATbleed
DX_CATcancer DX_CATchf DX_CATcopd DX_CATdm_lyte DX_CATembolus
DX_CATliver DX_CATmyocardial DX_CATneuro DX_CATother DX_CATpanc
DX_CATpna DX_CATrenal DX_CATresp DX_CATseizure DX_CATsepsis
LIMIT_CODESTATUSY PALL_PRE OPIOID_PRE CHRONIC_PAIN_PREY LOS
SMOKING_STATUSCURRENT SMOKING_STATUSFORMER SMOKING_STATUSNEVER
SMOKING_STATUSUNKNOWN
SOFA_DAY1 SOFA_MEDIAN21 IMY CHEST_TUBEY MV_EPISODES
MDFE_GRP/eventcode(cox)=1;
hazardratio MDFE_GRP;
baseline covariates=Inrisks out=Pred_all CIF=_ALL_/rowid=MDFE_GRP;
output out=surv survival=s atrisk=risk/method=pl ;
run;

/* add atrisk counts for table */
PROC SQL;
   CREATE TABLE WORK.pred3_all AS
   SELECT t1.MDFE_GRP,
          t1.time_to_event,
          t1.CIF,
          t1.StdErrCIF,
          t1.LowerCIF,
          t1.UpperCIF,
          t2.atr
      FROM WORK.PRED_all t1
           LEFT JOIN WORK.OUTCIF2 t2 ON (t1.MDFE_GRP = t2.MDFE_GRP) AND
(t1.time_to_event = t2.i);
QUIT;


ODS GRAPHICS/ RESET IMAGENAME = 'adjusted'
IMAGEFMT =PNG HEIGHT = 8in WIDTH = 11in ;
```

© 2024 Myers LC et al. *JAMA Network Open*.

```
ODS LISTING GPATH = "~/Output";


/* for atrisk table, only keep values in increment of 100 */
data pred3_all;
      set pred3_all;
      if mod(time_to_event,100) ne 0 then atr=.;
      if time_to_event<=365;
      run;
ODS GRAPHICS/ RESET IMAGENAME = 'adjusted_with_table'
IMAGEFMT =PNG HEIGHT = 8in WIDTH = 11in ;
ODS LISTING GPATH = "~/Output";
proc sgplot data=pred3_all noautolegend;
band x=time_to_event lower=LowerCIF upper=UpperCIF/
fillattrs=GraphData(transparency=0.8)
group=MDFE_GRP ;
styleattrs   datacolors=(black blue green red);
series x=time_to_event y=CIF / group=MDFE_GRP  name="series"
LEGENDLABEL = 'MDFE Group'  LINEATTRS = (THICKNESS = 3 pattern=1)
attrid=attrmapping;
styleattrs datacontrastcolors=(black blue green red);
xaxis label="Days from Discharge" values=(0 to 364 by 20);
yaxis grid  label="Probability of Opioid Script"
LABELATTRS=(Color=Black Family=Arial Size=10)
VALUEATTRS=(Family=Arial Size=10) ranges=(0-.5) valuesformat=percent6.1;
xaxistable atr/x=time_to_event class=mdfe_grp title="Number At Risk"
NOMISSINGCHAR
       labelattrs=(weight=bold)
       titleattrs=(weight=bold);
keylegend "series" / title="";
title 'Adjusted';
run;
```

© 2024 Myers LC et al. *JAMA Network Open*.

eTable 5. Beta Estimates of Covariates

Primary outcome is time to first opioid prescription with death as a competing risk

	coef	exp(coef)	se(coef)	z	Pr(>|z|)
AGE	-0.005455	0.99456	0.001884	-2.895	0.003787
GENDER (male)	-0.139534	0.869763	0.04671	-2.987	0.002815
RACE/Ethnicity - Black	0.222996	1.249816	0.099768	2.235	0.025407
RACE/Ethnicity - Hispanic	0.245021	1.277648	0.094687	2.588	0.009662
RACE/Ethnicity - Other	0.195134	1.215474	0.119618	1.631	0.102826
RACE/Ethnicity - White	0.293508	1.341124	0.081065	3.621	0.000294
Body mass index	0.005401	1.005415	0.002888	1.87	0.061447
Neighborhood deprivation index	0.003677	1.003684	0.025693	0.143	0.886203
Charlson Comorbidity Score	-0.048844	0.95233	0.042279	-1.155	0.24798
Chronic renal disease	0.1338	1.143164	0.076598	1.747	0.080675
Chronic pulmonary disease	0.157379	1.170439	0.065461	2.404	0.01621
Diabetes	0.092771	1.09721	0.082056	1.131	0.258234
Peripheral vascular disease	-0.019082	0.981099	0.074215	-0.257	0.797092
Chronic heart disease	0.025348	1.025672	0.072803	0.348	0.727712
Cerebrovascular disease	-0.097646	0.90697	0.07952	-1.228	0.219471
Myocardial infarction	0.023832	1.024119	0.079611	0.299	0.764667
Liver disease	0.089406	1.093525	0.080331	1.113	0.265719
Cancer	0.162413	1.176346	0.085554	1.898	0.057648
Acute respiratory failure category-Altered mental status	-0.330571	0.718513	0.420398	-0.786	0.431674
Acute respiratory failure category-cardiac arrest	-0.256392	0.773839	0.562927	-0.455	0.648778
Acute respiratory failure category-Arrhythmia	0.321183	1.378758	0.288663	1.113	0.265856
Acute respiratory failure category-Abdominal	0.39408	1.483019	0.306588	1.285	0.198663
Acute respiratory failure category-Bleed	-0.048392	0.95276	0.295055	-0.164	0.869723
Acute respiratory failure category-Cancer	0.920613	2.510828	0.350183	2.629	0.008565
Acute respiratory failure category-Heart failure	0.198827	1.219971	0.279702	0.711	0.477176
Acute respiratory failure category-COPD exacerbation	0.113111	1.119756	0.299857	0.377	0.706013
Acute respiratory failure category-Diabetes and electrolyte disturbance	0.460481	1.584836	0.321107	1.434	0.15156
Acute respiratory failure category-Pulmonary embolism	0.153531	1.165944	0.370721	0.414	0.67877
Acute respiratory failure category-Liver failure	0.170977	1.186464	0.328011	0.521	0.602189
Acute respiratory failure category-myocardial infarction	-0.053107	0.948279	0.288572	-0.184	0.853987
Acute respiratory failure category-Neurological condition	0.347371	1.415341	0.306714	1.133	0.257401

© 2024 Myers LC et al. *JAMA Network Open*.

	coef	exp(coef)	se(coef)	z	Pr(>|z|)
Acute respiratory failure category-Other	0.285179	1.33	0.265203	1.075	0.28223
Acute respiratory failure category-Pancreatitis	-0.034644	0.965949	0.357011	-0.097	0.922694
Acute respiratory failure category-Pneumonia	0.139912	1.150173	0.282891	0.495	0.620897
Acute respiratory failure category-Renal failure	-0.239195	0.787261	0.348799	-0.686	0.492859
Acute respiratory failure category-Acute respiratory failure not otherwise specified	0.032793	1.033337	0.260151	0.126	0.899688
Acute respiratory failure category-Seizure	-0.136421	0.872475	0.318362	-0.429	0.66828
Acute respiratory failure category-Sepsis	0.128723	1.137375	0.256076	0.503	0.615193
Code status limitation	0.078749	1.081933	0.067397	1.168	0.242635
Prior palliative care	-0.034342	0.966241	0.058823	-0.584	0.559341
Prior opioid use	1.033714	2.81149	0.052721	19.607	< 2e-16
Prior chronic pain diagnosis	0.590127	1.804218	0.052036	11.341	< 2e-16
Hospital length of stay	-0.003613	0.996394	0.001634	-2.211	0.027045
Former tobacco smoking	-0.205836	0.813967	0.081582	-2.523	0.011634
Never tobacco smoking	-0.377212	0.685771	0.082209	-4.588	4.47E-06
Unknown tobacco smoking	-0.170616	0.843145	0.091596	-1.863	0.062504
Sequential Organ Failure Assessment Score DAY1	-0.016224	0.983906	0.013088	-1.24	0.215113
Sequential Organ Failure Assessment Score, Mean	0.041568	1.042444	0.014673	2.833	0.004613
Invasive line	0.04662	1.047724	0.057974	0.804	0.421312
Chest tube	0.214674	1.239458	0.106347	2.019	0.043527
Number of mechanical ventilation episodes	-0.034942	0.965661	0.065132	-0.536	0.591625
Median Daily Fentanyl Equivalent dose Tercile 1 (0-67 mcg)	-0.003568	0.996438	0.080769	-0.044	0.964764
Median Daily Fentanyl Equivalent dose Tercile 2 (>67-700 mcg)	0.183034	1.200856	0.078548	2.33	0.019794
Median Daily Fentanyl Equivalent dose Tercile 3 (>700 mcg)	0.226587	1.254311	0.07946	2.852	0.00435

© 2024 Myers LC et al. *JAMA Network Open*.

eTable 6. Sensitivity Analyses of Exposure as Quartiles, Cumulative Dose Over Hospitalization, and Highest Hourly Dose

The outcome for all sensitivity analyses was first opioid script, We performed a cause-specific Cox proportional hazard regression with death as a competing risk.

Median daily fentanyl equivalent exposure as <u>quartiles</u>

	**Unadjusted HR (95%CI)**	**Adjusted HR (95%CI)**
Quartile 1 (0-40 mcg)	0.99 (0.84, 1.17)	0.95 (0.81, 1.13)
Quartile 2 (>40-200 mcg)	1.33 (1.14, 1.56)	1.21 (1.03, 1.42)
Quartile 3 (>200-1000 mcg)	1.38 (1.18, 1.62)	1.24 (1.06, 1.45)
Quartile 4 (>1000 mcg)	1.65 (1.41, 1.92)	1.34 (1.15, 1.58)

Cumulative dose of fentanyl equivalent over entire hospitalization (including both drips and pushes) exposure

	**Unadjusted HR (95%CI)**	**Multivariable HR (95%CI)**
Tercile 1 (0-400 mcg)	1.16 (0.96, 1.39)	1.10 (0.92, 1.33)
Tercile 2 (>400-2833.5 mcg)	1.53 (1.28, 1.84)	1.34 (1.11, 1.61)
Tercile 3 (>2833.5 mcg)	1.82 (1.53, 2.18)	1.48 (1.23, 1.79)

Highest hourly dose of fentanyl equivalent over the first episode of mechanical ventilation exposure

	**Unadjusted HR (95%CI)**	**Multivariable HR (95%CI)**
Tercile 1 (0-50 mcg)	1.18 (1.02, 1.37)	1.09 (0.94, 1.27)
Tercile 2 (>50-125 mcg)	1.37 (1.18, 1.59)	1.21 (1.04, 1.41)
Tercile 3 (>125 mcg)	1.49 (1.28, 1.73)	1.26 (1.08, 1.48)

© 2024 Myers LC et al. *JAMA Network Open*.

eTable 7. Additional Analyses Including Interactions Terms

The following are 3 interactions between median daily fentanyl equivalent (MDFE) terciles for the primary outcome of time until first opioid prescription with death as a competing risk and censoring for loss of KPNC membership:

1. **Opioid-related diagnosis code or filled opioid prescription in 1 year before hospitalization by MDFE tercile**

There were 3,306 patients who had an opioid-related diagnosis code or filled opioid prescription in the 1 year prior to hospitalization.

	**HR**	**Lower CL**	**Upper CL**	**P-value**
Prior opioid by MDFE Tercile 1 (0-67 mcg) vs. no opioid	0.90	0.65	1.24	0.515
Prior opioid by MDFE Tercile 2 (>67-700 mcg) vs. no opioid	0.85	0.62	1.16	0.304
Prior opioid by Tercile 3 (>700 mcg) vs. no opioid	1.14	0.83	1.57	0.420

2. **Chronic pain diagnosis in 1 year before hospitalization by MDFE tercile**

There were 1,566 patients who had a diagnosis of chronic pain in the 1 year prior to hospitalization.

	**HR**	**Lower CL**	**Upper CL**	**P-value**
Chronic pain by MDFE Tercile 1 (0-67 mcg)	0.90	0.65	1.26	0.544
Chronic pain by MDFE Tercile 2 (>67-700 mcg)	0.90	0.65	1.23	0.492
Chronic pain by MDFE Tercile 3 (>700 mcg)	1.04	0.77	1.42	0.788

3. **Palliative care referral in 1 year before hospitalization by MDFE tercile**

There were 768 patients who had been referred to palliative care in the 1 year prior to hospitalization.

	**HR**	**Lower CL**	**Upper CL**	**P-value**
Pall care by MDFE Tercile 1 (0-67 mcg)	0.94	0.67	1.31	0.702
Pall care by MDFE Tercile 2 (>67-700 mcg)	1.19	0.86	1.65	0.294
Pall care by MDFE Tercile 3 (>700 mcg)	0.99	0.71	1.37	0.931

© 2024 Myers LC et al. *JAMA Network Open*.

eFigure 2. Overlap of Patients Who Meet Various Definitions of Persistent Opioid Use

Our definition of persistent opioid persistent use was the ratio of number of opioid prescriptions to number of months of KPNC healthcare plan enrollment ≥0.8 (top blue circle). The Venn diagram below shows the overlap of this definition with the 2 other definitions used in the perioperative literature. Most patients meeting our definition of persistence (n=634, 83.9%) also met criteria for the other 2 definitions (≥10 prescriptions in the 1-year post-discharge period or ≥3 months of prescriptions filled).

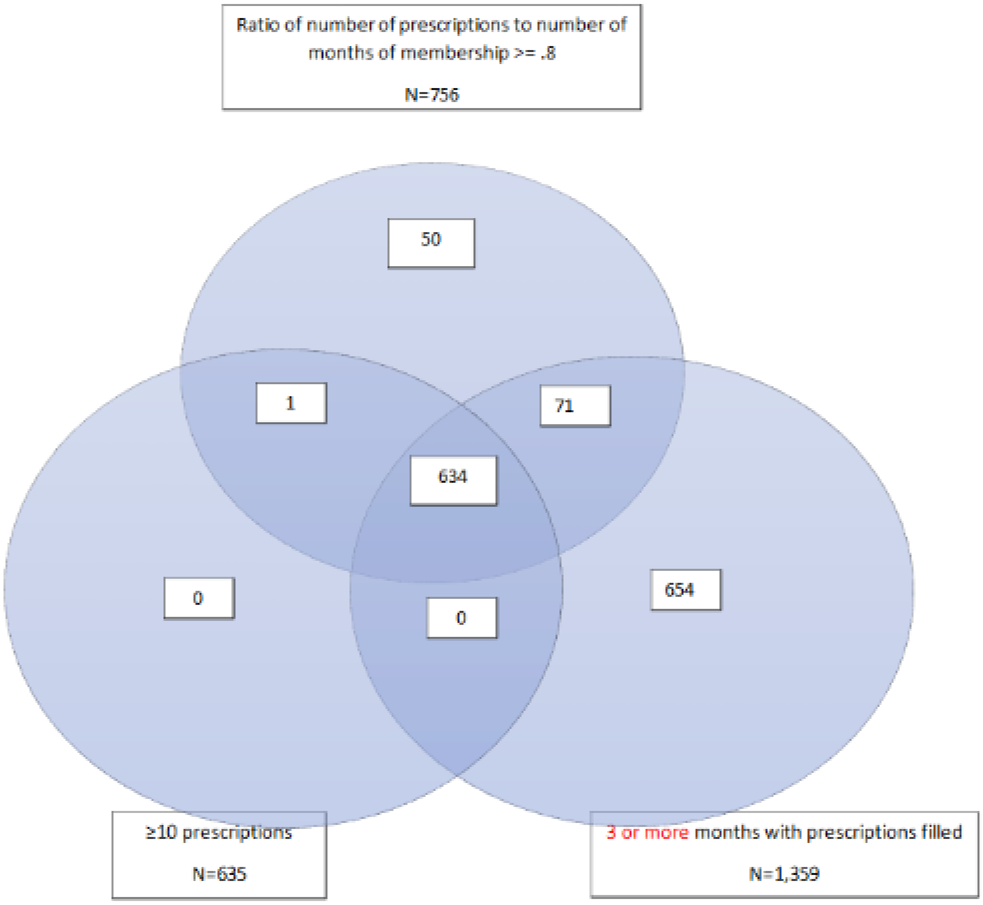

© 2024 Myers LC et al. *JAMA Network Open*.
