# Supplementary material for: Intravenous Opioid Administration During Mechanical Ventilation and Use After Hospital Discharge
Source: JAMA Netw Open. 2024 Jun 14;7(6):e2417292. doi: 10.1001/jamanetworkopen.2024.17292 (PMC11179130; doi:10.1001/jamanetworkopen.2024.17292)
Supplement: Supplement 2. — Data Sharing Statement [file jamanetwopen-e2417292-s002.pdf]

Data Sharing Statement

Myers. Intravenous Opioid Administration During Mechanical Ventilation and Use After Hospital Discharge. *JAMA Netw Open*. Published June 14, 2024.
doi:10.1001/jamanetworkopen.2024.17292

Data
Data available: No

Additional Information
Explanation for why data not available: The datasets generated and/or analyzed during the current study are not publicly available due to their being the property of Kaiser Foundation Health Plan, Inc., but are available to interested collaborators in the context of a formal collaboration approved by the Kaiser Permanente Northern California Institutional Review Board for the Protection of Human Subjects.
